# Unraveling Alpha-Gal Syndrome: A Case Study of a Rare Meat Allergy

**DOI:** 10.7759/cureus.65437

**Published:** 2024-07-26

**Authors:** Riya Patel, Anirudh Jaglan, Clarisa Aguileraserna, Krutarth Pandya, Lawrence Goldstein

**Affiliations:** 1 Internal Medicine, Western Reserve Health Education/NEOMED, Warren, USA; 2 Internal Medicine, Ross University School of Medicine, Bridgetown, BRB; 3 Internal Medicine, Cleveland Clinic, Cleveland, USA; 4 Pulmonary and Critical Care Medicine, Western Reserve Health Education/NEOMED, Warren, USA

**Keywords:** rare allergy, alpha-gal allergy, alpha-gal syndrome, food allergy quality of life, meat allergy

## Abstract

Allergies are a very common pathology and their manifestations consist of a spectrum of presentations, ranging from minimal discomfort like a runny nose to lethal reactions like anaphylaxis and death. Meat allergy is not a very common form of allergy, even though there is a relatively high level of meat consumption. One of the rare forms of non-primate mammalian meat allergy is alpha-gal syndrome (AGS). It is related to IgE antibodies specific for galactose-α-1,3-galactose (α-Gal). It is triggered in sensitized individuals due to multiple bites of lone star tick bites (*Amblyomma americanum*). Here we present a 63-year-old male with a complaint of recurrent hives and tongue swelling, developed recently after traveling to Twinsburg, OH. There is no significant history of any allergic conditions. Vital signs were stable with a normal physical examination. The patient had normal routine labs including eosinophil count, thyroid-stimulating hormone (TSH), iron panel, and negative HIV. Allergy testing showed normal total IgE but increased levels of IgE for allergens like beef, lamb, and pork (18.4, 6.71, and 7.62 respectively) and greatly increased levels of IgE for alpha-gal (42.7). Sensitization to alpha-gal can cause delayed allergic symptoms upon consuming various non-primate mammalian types of meat, particularly beef, pork, and lamb. Conditions like AGS are rare and can be missed as an initial diagnosis in many patients. A high degree of vigilance is required to diagnose such conditions.

## Introduction

Allergy to various types of meat is uncommon even in developed countries despite a relatively high level of meat consumption. Childhood meat allergy is usually associated with atopic dermatitis and is usually outgrown during the first years of life. Meat allergy is not restricted to children but can also develop in adults [1]. Alpha-gal syndrome (AGS) is a rare kind of meat allergy, usually associated with tick bites. It is a potentially life-threatening immunoglobulin E (IgE)-mediated hypersensitivity to galactose-alpha-1,3-galactose, an oligosaccharide found in most non-primate mammalian tissue and other mammalian products like milk and some of the pharmaceuticals [2,3]. Recent visits to a tick-infested area and allergic symptoms after meals are certain aspects of the patient’s history on presentation that point toward the diagnosis of AGS. Definitive diagnosis is established by the detection of serum alpha-gal specific IgE for red meat allergy. Furthermore, when an oral food challenge, if performed, shows positive, it gives a confirmative diagnosis [4]. In this case, we present a 63-year-old male with a history of recent travel to a tick-infested area and developing an allergic reaction to meat later on. Diagnostic studies evidence meat allergy with an elevated IgE for red meat. Thus, AGS is a rare kind of meat allergy that manifests after multiple tick bites and can present in any age group.

## Case presentation

A 63-year-old male presented to the outpatient clinic with chief complaints of recurrent hives and tongue swelling. The patient had a past medical history of hypertension, hyperlipidemia, coronary artery disease, supraventricular tachycardia, diabetes mellitus type 2, benign prostate hyperplasia, diverticulosis, degenerative disc disease, and iron deficiency anemia as listed in Table 1. Past surgical history included a history of tonsillectomy. As for the social history, he had never smoked, occasionally drank alcohol, and denied any recreational drug use. He lived in Tennessee but had moved to Ohio two years before this incident. The patient had a remote memory of being allergic to lisinopril. He was up to date with his influenza vaccination but did not take any other vaccines. His routine medications included hydrochlorothiazide, metoprolol, atorvastatin, fenofibrate, omega 3 fatty acid, aspirin, clopidogrel, glyburide, metformin, and jardiance, as listed in Table 2.

**Table 1:** Past medical history of the patient on the day of presentation The table includes the medical problems that the patient had in the past or is being currently managed for.

Past Medical History
Hypertension
Hyperlipidemia
Diabetes mellitus type 2
Benign prostate hyperplasia
Diverticulosis
Supraventricular tachycardia
Coronary artery disease
Iron deficiency anemia
Degenerative disc disease

**Table 2:** Home medications taken by the patient The table includes the list of the medications taken by the patient at home before this visit to the physician.

Medications
Hydrochlorothiazide
Metoprolol
Atorvastatin
Fenofibrate
Omega-3 fatty acid
Aspirin
Clopidogrel
Glyburide
Metformin
Jardiance

The patient had a recent travel history to Twinsburg when he broke out into hives but did not have the slightest idea of the cause of that symptom. During his current visit, he had a BMI of 29.57 kg/m^2^ and the rest of the vital signs were within normal limits. The review of his systems was positive for hives and tongue swelling. The patient denied fever, chills, night sweats, weight gain/loss, dizziness, confusion, chest pain, palpitations, abdominal pain, nausea, vomiting, diarrhea, or lower extremity edema. Physical examination was normal with no rashes or hives over the skin. Laboratory parameters, as listed in Table 3, included a normal white blood cell count of 7.3, hemoglobin of 9.9, and normal electrolytes including sodium, potassium, calcium, and chloride at 140,4, 10.7, and 24 respectively. Kidney function was normal as well with a creatinine of 1.1 and a glomerular filtration rate (GFR) of greater than 60. Liver function was also normal with aspartate aminotransferase (AST) and alanine aminotransferase (ALT) of 12 and 15 respectively, along with alkaline phosphatase (ALP) of 85. The patient had a known history of diabetes mellitus and his hemoglobin A1C was measured to be 9.6. He had some iron deficiency anemia with a low iron of 41. Thyroid stimulating hormone was normal and he was found to be non-reactive to HIV antibody as well as hepatitis C antibody.

**Table 3:** Laboratory values of the patient on the day of presentation The table includes the results of the blood drawn on the day of the presentation which gives more insight into the differentials. WBC: White Blood Cell; RBC: Red Blood Cell; MCV: Mean Corpuscular Volume; Co_2_: Bicarbonate; BUN: Blood Urea Nitrogen; GFR: Glomerular Filtration Rate; ALP: Alkaline Phosphatase; ALT: Alanine Amino Transferase; AST: Aspartate Amino Transferase; HbA1C: Hemoglobin A1C; TSH: Thyroid Stimulating Hormone; TIBC: Total Iron Binding Capacity; HIV: Human Immunodeficiency Virus

Parameter	Result	Reference Range
WBC	7.3	4.5-11
RBC	4.13	3.8-5.8
Hemoglobin	9.9	12.5-16.5
MCV	77.5	80-99.9
Platelets	554	130-450
Neutrophil%	60.1	43-80
Lymphocyte%	28.8	20-42
Monocyte%	6.3	2-12
Eosinophil%	3.6	0-6
Lymphocyte absolute	2.11	1.5-4
Monocyte absolute	0.46	0.1-0.95
Sodium	140	132-146
Potassium	4	3.5-5
Chloride	24	98-107
Co_2_	24	22-29
Anion Gap	14	7-16
Glucose	318	74-99
BUN	26	6-23
Creatinine	1.1	0.7-1.2
GFR	>60	>60
Calcium	10.7	8.6-10.2
Total protein	7.6	6.4-8.3
Total bilirubin	<0.2	0-1.2
ALP	85	40-129
ALT	15	0-40
AST	12	0-39
HbA1C	9.6	4-5.6
TSH	1.33	--
Iron	41	59-158
TIBC	430	250-450
Iron saturation	10	20-50
Ferritin	11	--
HIV antibody	Non-reactive	--
Hepatitis C antibody	Non-Reactive	--

Along with the routine labs, allergen screening including immunoglobulin levels was ordered (Table 4). These labs showed that the levels of Immunoglobulin, Ig E, were elevated for beef (18.40), lamb (6.71), and pork (7.62) allergens in the food. The alpha-gal allergen IgE (42.7) was seen to be magnanimously elevated as well. This shows that this patient was developing the hives due to an allergy to beef, lamb, or pork protein allergens in the food. This is a rare allergic reaction, and the patient had a recent visit to Twinsburg, Ohio which has a high tick infestation. This kind of allergic reaction usually is associated with multiple tick bites. Thus, travel history is also a crucial part of making the diagnosis in cases like this. Though AGS is a rare meat allergy, it should be considered as a differential when evaluating sudden allergic reactions in patients with recent travel to a tick-infested area, especially those who have concurrent atopic dermatitis, gelatin allergy, or cow’s milk allergy as well as those with blood groups A and O.

**Table 4:** Laboratory values for the allergen panel test for meat allergy IgE: Immunoglobulin E The table shows the increased values of Immunoglobulin E as compared to the reference normal range, for various varieties of meat, thus proving the diagnosis of alpha-gal syndrome.

Parameter	Result	Reference Range
Immunoglobulin IgE	194	≤214
Allergen, Food, Beef	18.40	≤0.34
Allergen, Food, Lamb	6.71	≤0.34
Allergen, Food, Pork	7.62	≤0.34
Allergen, Food, Alpha-Gal, IgE	42.70	≤0.09

The patient was advised to always keep an Epinephrine pen with him in addition to avoiding meat and meat products, dairy products, and gelatin in food.

## Discussion

The carbohydrate moiety galactose-alpha-1,3-galactose (alpha-gal/α- gal) is abundantly expressed in cells and tissues of all mammalian species except primate mammals (i.e., humans, chimpanzees, and old-world monkeys) [5,6]. When Karl Landsteiner defined the ABO system, he described a structurally similar “B-like” glycoprotein that at the time, he reported all immunocompetent individuals had agglutinating antibodies to [7]. IgE to α- gal was first described in 2006 with two anaphylactic reactions to the monoclonal antibody, cetuximab, in Arkansas, USA. The discovery of α-gal specific IgE in 2008 was new and in 2009, these antibodies were shown to elicit an anaphylactic reaction not only to this drug but also to red meat consumption of certain mammals [6]. Mammalian meat allergy was thought to be restricted to children, primarily ones with atopic dermatitis or cow’s milk allergy, but it has now been shown to be equally present in adults. AGS was the proposed term to describe this disease because of the three features of the allergy: the food allergy with a type-1 delayed reaction, the drug allergic reaction, and the allergic reaction to tick bites [6,8]. This novel syndrome was investigated by the National Institute of Allergy and Infectious Diseases, Division of Allergy, Immunology and Transplantation in 2018. The intended purpose of the workshop by this institution was to review and discuss the mechanism of IgE response after tick bites and the mechanism and significance of the delayed reaction, plus the severity of reactions to drugs.

In susceptible individuals, multiple tick bites appeared to result in sensitization to the carbohydrate allergen α-gal. The relationship between tick bites and the sensitization to the oligosaccharide was first suggested in Australia and then in the US after findings of IgE antibodies to α-gal were seen in 20% of patients and controls in Tennessee, Virginia, North Carolina, Arkansas, and southern Missouri. This finding posed the question as to why individuals in these areas had a higher rate of allergic reactions to mammalian meat products. In the US, the distribution matched up with areas with a high prevalence of Rocky Mountain Spotted Fever (RMSF). Commins and Platts-Mills provided evidence that showed the rise in IgE antibodies to α- gal after tick bites by analyzing histories of tick bites in patients and through serological assays using Amblyomma americanum and Dermacentor variabilis extracts [9,10]. This was further supported by evidence provided by a group in Stockholm demonstrating the presence of α-gal in the gut of the tick and similar findings in southern Sweden, but this is not to say that all ticks can cause sensitization [7].

Patients sensitized to α-gal reported delayed symptoms in a wide range of mammalian meats, especially beef, pork, and lamb. This feature of AGS is pathognomonic and has been reported in many case reports and challenge studies all over the world. Notably, a collection of case reports put together by Dr. Jeppe in Germany demonstrated that patients who had sensitization to α-gal showed an immediate reaction, within 1 hr, to pork kidney, but a time-delayed reaction when muscle meat was consumed [8]. The same delayed reaction was noted by physicians in Australia, Sweden, Spain, Japan, and Korea [7,9]. Understanding the mechanism of the delayed reaction as well as the role ticks play in mammalian meat allergy remains unclear. The need for further research is necessary but challenging due to the delayed onset of reactions requiring monitoring of patients as well as the need for sensitive testing for accurate diagnosis. The rarity of this syndrome with an unorthodox allergic reaction also poses a challenge for physicians in appropriately diagnosing, advising, and managing patients. This area is of great importance due to the severe anaphylactic reactions when cetuximab is administered. The workshop put together by the National Institute of Allergy and Infectious Diseases on understanding IgE-mediated mammalian meat allergy recommended that future research focus on epidemiology and observational research, identifying specific tick species, identifying early symptomatology, and areas of pathophysiologic research [5]. Patients are usually advised to avoid meat and meat products, dairy products, and gelatin in food as well as cetuximab, horse and snake-derived anti-venom therapy, and bovine or porcine-derived thyroid hormone supplements. Patients who remain symptomatic post these measures may be advised to avoid heparin products, pancreatic enzymes, gel capsules, and vaccines with gelatin-like MMR as well as bovine and porcine heart valves [4].

## Conclusions

Thus, this case presents the need for detailed history taking, including travel history, allergies, and eating habits, to keep an elevated level of suspicion and due vigilance for the patients who present with either chronic urticarial or allergic reactions after consumption of mammalian meat products as well as known tick-bite history, once all other causes have been ruled out.
